# Primary monophasic synovial sarcoma presenting as a pulmonary mass: a case report

**DOI:** 10.1186/1752-1947-2-18

**Published:** 2008-01-24

**Authors:** Charalampos M Mermigkis, Antony Kopanakis, George Patentalakis, Vlassis Polychronopoulos, Michael Patentalakis

**Affiliations:** 1 Third Pulmonary Department, Sismanoglio General District Hospital, Athens, Greece

## Abstract

**Introduction:**

Primary pulmonary synovial sarcoma is an extremely rare tumor with only few case reports in the literature.

**Case presentation:**

A healthy 67-year-old woman was admitted for investigation of a pulmonary mass found on a routine X-ray. She had a history of breast cancer diagnosed and treated 13 years previously with left mastectomy followed by adjuvant endocrine therapy. No progression of the disease was reported. Thoracic computer tomography disclosed a soft-tissue mass in the lower lobe of the left lung arising in the vicinity of the pleura. No abnormal lymph nodes were noted. Further work-up for metastases was negative. Subsequently, the lower lobe of the left lung was removed and the diagnosis was a monophasic synovial sarcoma.

**Conclusion:**

The diagnosis of monophasic primary pulmonary synovial sarcoma requires clinical, imaging and immunohistochemical investigation to exclude alternative primary sources. The treatment of choice is excision (lobectomy or pneumonectomy), which in most of cases is helpful for diagnosis. The prognosis is usually poor.

## Introduction

Primary synovial sarcoma of the lung is an extremely rare tumor [1,2] and seems to be strongly related to cigarette smoking [3]. A definitive diagnosis requires detailed immunohistochemical staining [4,5], as well as clinical and imaging investigation to exclude alternative primary sources.

We describe a case of an asymptomatic, 67-year-old, non smoking woman with a primary monophasic synovial sarcoma presenting as a left lower lobe pulmonary mass.

## Case presentation

A 67-year-old woman was referred for investigation of a peripheral opacity in the left lung lower lobe, which was discovered incidentally on a chest radiograph. Thirteen years earlier she had undergone a radical left-sided mastectomy for breast cancer, followed by a three-year course of adjuvant endocrine therapy with tamoxifen. No adjuvant radiation therapy or chemotherapy was given after the mastectomy. Until the time of presentation, no evidence of disease recurrence had been found.

The patient was a housewife, did not smoke or abuse alcohol, had no known toxic exposures and denied any family history of malignancies. She was in good general health and well nourished. Physical examination was negative apart from a post-surgical chest scar. The results of blood tests, standard biochemical tests, urine analysis, c-ANCA, p-ANCA, tumor markers and arterial blood gas analysis were normal.

**Figure 1:**
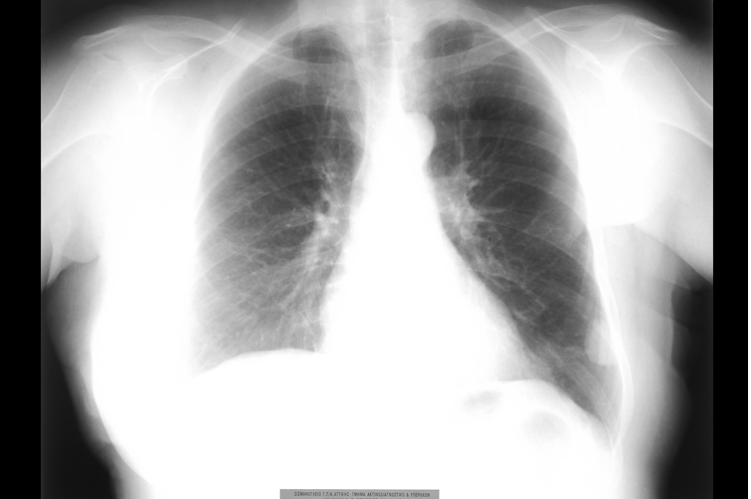
Chest Radiograph.

**Figure 2:**
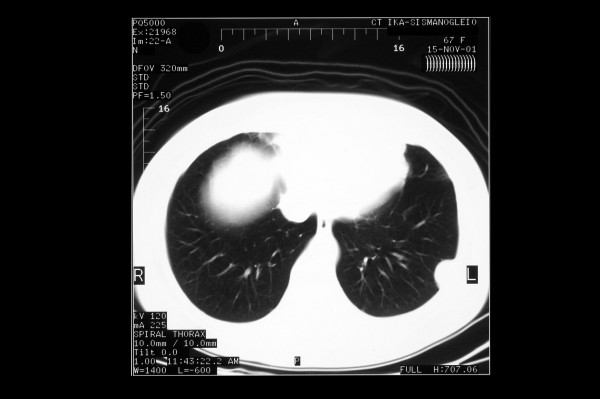
Chest computed tomography (CT).

The chest radiograph (Figure 1) revealed a well-demarcated, non-cavitating, 3 cm in diameter peripheral opacity in the left lower lobe, with obtuse angles between the lesion and the pleura, and no other abnormalities. The lesion was not apparent on a chest radiograph performed one year previously. Chest computed tomography (CT), (Figure 2) confirmed a 3 × 4 cm, oval-shaped, well-delineated peripheral mass in the left lower lobe, abutting the pleura, homogenous in attenuation and no evidence of mediastinal or axillary adenopathy. CT of the brain and abdomen revealed no pathological findings. Pulmonary function studies were normal. Bronchoalveolar lavage and bronchial brushing specimens, obtained during bronchoscopy, were negative for malignancy. The CT-guided fine needle aspirate from the mass revealed clusters of neoplastic cells of unclear affiliation, strongly positive on immunostaining for vimentin and negative for carcino-embryonic antigen (CEA) and epithelial membrane antigen (EMA).

**Figure 3:**
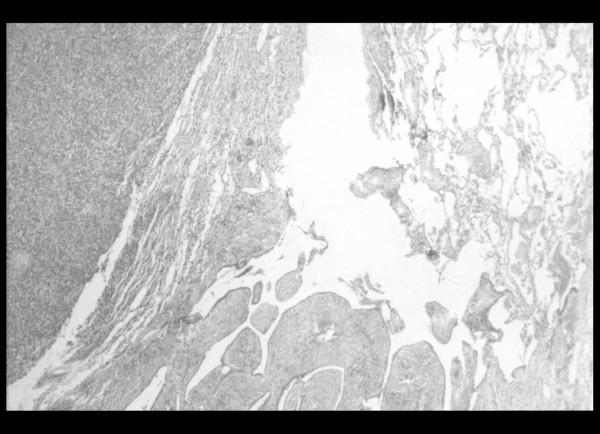
Section of the lung lesion (Hematoxylin and Eosin stain × 40): In this section, the morphology of a spindle cell neoplasm is apparent, with slit-like spaces and normal adjacent lung parenchyma.

**Figure 4:**
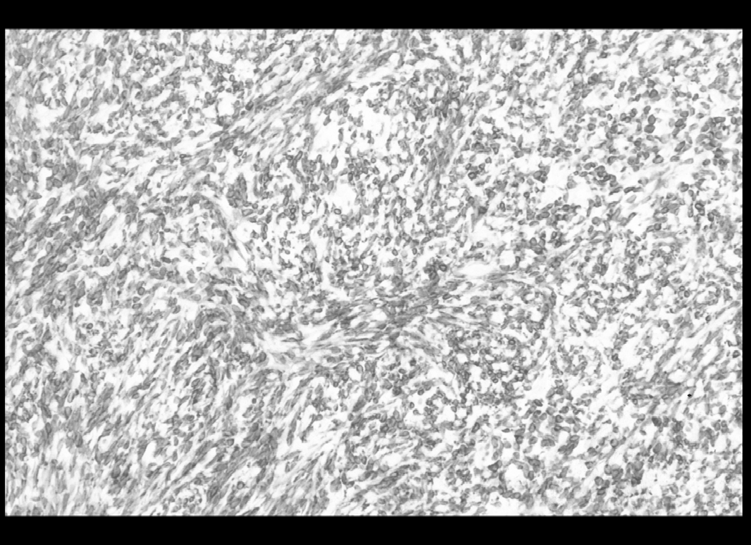
CD99 and Bcl-2 positive spindle-like neoplastic cells (immunohistochemical stain, × 100).

Finally, a left thoracotomy was performed, revealing a tumor in the outer part of the left lower lobe, which did not infiltrate the visceral pleura. Subsequently, a left lower lobectomy with typical excision of the satellite lymph nodes was completed. Pathological examination revealed a well-circumscribed, whitish, soft tumor measuring 2.5 × 3 × 2 cm. Microscopic examination showed a spindle cell neoplasm, with slit-like spaces and normal adjacent lung parenchyma (Figure 3). Immunohistochemical staining was positive for CD99 and Bcl-2, while staining for EMA, CK19, CK17, BerEP4, S-100 and thrombomodullin was negative. The above findings were compatible with a monophasic spindle cell type synovial sarcoma in the lung (Figure 4). The lymph nodes showed lesions of reactive lymphadenitis, and the specimens of pleura showed edema and low-grade inflammation.

## Discussion

Synovial sarcoma (SS) is a rare and well-established mesenchymal tumor, accounting for approximately 10% of all soft tissue tumors [6,7]. SS typically presents in adolescents and young adults, most commonly in the soft tissues of the extremities (especially near large joints), but neck, lung, heart, mediastinum and abdominal wall sites have been reported [6]. The term "synovial" sarcoma was given because of the synovial differentiation of the tumor that is believed to originate from multipotential mesenchymal cells. SS is a highly aggressive tumor affecting males more often than females, and seems to be strongly related to cigarette smoking [3,7].

The generally accepted histological subtypes of synovial sarcoma are biphasic, monophasic spindle cell or fibrous, monophasic epithelial and poorly differentiated subtypes [8]. Among them the monophasic neoplasm subtype occurs most often in the lung [9]. The biphasic type is easily diagnosed based on the presence of both epithelial and spindle cell components. The monophasic type is difficult to diagnose, because it has a uniform spindle cell pattern; thus it may be confused with other malignant spindle cell neoplasms, such as fibrosarcoma, hemangiopericytoma, leiomyosarcoma and spindle cell carcinoma or carcinosarcoma.

Immunohistochemistry plays a crucial role in the diagnosis of SS, especially in monophasic type cases. Most synovial sarcomas show immunoreactivity for cytokeratins and epithelial membrane antigen (EMA). Furthermore, 30% of them are protein S-100 positive, 60–70% CD 99 positive and 75–100% Bcl-2 positive [10].

Cytogenetics also play an important role since both the monophasic and the biphasic form are characterised by a reciprocal chromosomal translocation (x;18) (p 11.2;q11.2). The translocation fuses the *SYT *gene from chromosome 18 to either of two homologous genes at Xp11, *SSX1 *or *SSX2 *[11,12]. Despite its high sensitivity, molecular testing is not required if the diagnosis of synovial sarcoma is certain or probable on the basis of clinical, histological, and immunohistochemical evaluations [12,13].

Pulmonary sarcomas are rare accounting for less than 0.5% of lung cancers and most malignant mesenchymal tumors of the lung are metastases of a primary tumor from elsewhere [2,6]. Leiomyosarcomas, fibrosarcomas and hemangiopericytomas are the most common types of primary pulmonary sarcomas [2]. Primary pulmonary SS are extremely rare with only few case reports in the literature. The diagnosis can be established only after clinical and imaging investigation to exclude alternative primary sources [1,2,8].

Two thirds of the reported cases primary pulmonary SS were centrally located and associated with complaints of postobstructive pneumonia (cough, dyspnea, fever) and hemoptysis. Peripheral tumors are less common and usually asymptomatic, but may infiltrate adjacent tissues (pleura, thoracic wall, and mediastinum) or give rise to distant metastases (adrenals, brain, and spinal cord) [1,2]. It is noteworthy that in our patient no infiltration of adjacent tissues was found.

Sputum examination is rarely helpful and bronchoscopy is diagnostic in 40–60% of cases. Differential diagnosis includes bronchogenic carcinoma, different metastatic lesions, mesothelioma, lymphoma, Wegener's granulomatosis, pyogenic abscess, intrapulmonary hematoma, rheumatoid nodules, histoplasmosis and coccidiomycosis. Lung SS frequently metastasizes to regional lymph nodes (hilar or mediastinual lymphadenopathy) as well as to distant sites (adrenals, brain, bone marrow). Owing to its rarity and the paucity of data regarding its natural history, there are no guidelines for optimal treatment. Therefore, current treatment includes surgical resection (lobectomy or pneumonectomy) followed by adjuvant radiotherapy or chemotherapy [2,6,14]. Prognosis is related to the disease stage and is usually poor. In available case series and reports, the five-year survival ranges between 36% and 76% [2]. Our patient underwent a lobectomy without adjuvant chemotherapy. Five years and seven months after operation, the patient remains asymptomatic and without any evidence of disease recurrence.

## Conclusion

Primary pulmonary synovial sarcoma is an extremely rare neoplasm. Clinical and imaging investigation is necessary to exclude alternative primary sources, while a definitive diagnosis requires detailed immunohistochemical staining (cytokeratins, vimentin, S100, CD20, CD99, Bcl-2 and other markers). A balanced chromosomal translocation, t(X;18) (p11.2;q11.2), is found in the majority of synovial sarcomas resulting in a chimeric transcript, SYT-SSX, the role of which is so far unclear. Surgical excision with clear margins and possibly adjuvant chemo-radiotherapy is the currently accepted treatment.

## Competing interests

The author(s) declare that they have no competing interests.

## Authors' contributions

CHM participated in the design of the study and performed the statistical analysis. AK participated in the design of the study and performed the statistical analysis. GP carried out the molecular genetic studies, participated in the sequence alignment and drafted the manuscript. VP conceived of the study, and participated in its design and coordination and helped to draft the manuscript. MP conceived of the study, and participated in its design and coordination and helped to draft the manuscript.

## Consent

The authors confirm that writteninformed patient consent was sought and granted for details of this case report to be published.
